# Development and Characterization of Litchi Seed Polyphenol-Loaded Camellia Oil Oleogels Based on Citrus Pectin–Lecithin Emulsion Templates

**DOI:** 10.3390/foods15173014

**Published:** 2026-08-27

**Authors:** Xinglong Zhao, Hong Niu, Xiujuan Fu, Siwei Chen, Hao Liu, Guozheng Nie, Lihua Hu, Hui Lei, Dan Zhang, Yu Luo

**Affiliations:** 1 School of Pharmacy, Southwest Medical University, Luzhou 646000, China; xilzhao@163.com (X.Z.); dawn123454@swmu.edu.cn (H.N.); uoyu@sina.com (X.F.); yili611@163.com (S.C.); h_lewis@126.com (H.L.); gznie0318@163.com (G.N.); 2 Sichuan Xishu Jiujin Modern Traditional Chinese Medicine Co., Ltd., Luzhou 646100, China; lihuaehu@163.com

**Keywords:** litchi seed polyphenols, oleogels, camellia oil, citrus pectin, emulsion template

## Abstract

Traditional plastic fats are rich in saturated and trans fatty acids, which can lead to chronic diseases. In contrast, camellia oil and litchi seed polyphenols are valuable natural functional ingredients. This work aimed to construct stable camellia oil oleogels using citrus pectin as the gel-forming component, lecithin as the emulsifier for constructing the emulsion template, and litchi seed polyphenols as both auxiliary gel-strengthening and antioxidant components. The oleogels were fabricated via emulsion templating combined with freeze-drying and shear forming. Effects of oil–water ratio and pectin concentration on microstructure, texture, rheology, oil-holding and thermal stability were investigated, with antioxidant activity evaluated by DPPH radical scavenging activity, acid value, and peroxide value. The optimal emulsion was obtained at oil–water ratio 5:5 and 4.0% pectin with uniform W/O droplets of 3.90 ± 0.24 μm. Oleogels with 3.0% pectin showed the best comprehensive performance, presenting dense network, favorable thermal stability and superior oil-binding capacity, along with a DPPH scavenging rate of 83.07 ± 0.06%. FTIR suggested that the oleogel network was primarily stabilized by non-covalent interactions without the formation of new chemical bonds. The prepared oleogels exhibited stable physicochemical properties and enhanced DPPH radical scavenging activity and oxidative stability, showing great potential in functional foods and topical delivery systems.

## 1. Introduction

With the escalating prevalence of metabolic syndromes such as cardiovascular disease and obesity, the reduction in saturated fats and the elimination of trans-fatty acids in processed foods have become a global priority [1]. According to the World Health Organization (WHO), more than 2.5 billion adults worldwide were overweight in 2022, including over 890 million living with obesity, and cardiovascular disease remains the leading cause of death globally, highlighting the urgent need to develop healthier lipid-based food systems [2]. Oleogels, characterized as three-dimensional networks that entrap liquid vegetable oils into a solid-like structure, offer a promising alternative to traditional plastic fats [3]. Unlike chemical interesterification or modification, which often suffer from complex product compositions and challenging purification processes, oleogelation provides a more straightforward and physical route to structure liquid oils [4].

The formation of oleogels primarily relies on physical cross-linking through non-covalent interactions, such as hydrogen bonding and Van der Waals forces, to construct a stable structural framework [5]. Among various fabrication strategies, the emulsion-templated approach stands out due to its mild processing conditions, rendering exceptional capability for the encapsulation of heat-sensitive bioactive compounds [6]. The phase behavior of emulsion templates is strongly influenced by the relative volume fraction of oil and aqueous phases, as well as the interfacial stabilization capacity of emulsifiers. Variations in phase composition may induce transitions between oil-in-water and water-in-oil structures, which are critical considerations for designing stable templates for oleogel preparation [7]. In addition, surfactant–biopolymer interactions have been reported to improve emulsion stability by enhancing interfacial protection and steric stabilization. Amphiphilic surfactants can rapidly adsorb at oil–water interfaces, while polysaccharides contribute to the stabilization of the interfacial layer and continuous phase [8]. Furthermore, recent evidence suggests that the interaction between polyphenols and polysaccharides (e.g., pectin) can significantly reinforce the gel network through cross-linking, while simultaneously providing a synergistic antioxidant effect between the lipid phase and the polyphenolic antioxidants [9].

Camellia oil, often referred to as “Oriental Olive Oil,” is highly valued for its high oleic acid content and potent anti-inflammatory properties [10]. Recent studies have explored the structuring of liquid vegetable oils through various oleogelation strategies, including wax-based, polymer-based, and emulsion-templated approaches. These studies demonstrate the potential of oleogels for converting liquid oils into structured lipid materials; however, multifunctional oleogels based on camellia oil combined with natural polyphenol incorporation remain relatively limited [11]. In recent years, camellia oil has attracted increasing attention as one of the most important woody edible oils in China, with an annual production exceeding 1 million tons, reflecting its growing industrial and nutritional significance [12]. However, its liquid nature limits its application in food formulations that require structural integrity. Concurrently, litchi seeds—a common agricultural byproduct—are rich in litchi seed polyphenols (LSP), which exhibit significant antioxidant activities yet suffer from poor environmental stability [13]. Oil gelation can protect these unstable polyphenols. Compared with commonly investigated plant polyphenols, litchi seed polyphenols are derived from an underutilized agro-industrial byproduct, providing an opportunity to simultaneously improve the functional value of oleogels while promoting the high-value utilization of litchi processing residues [14]. However, although previous studies have investigated protein- or polysaccharide-based oleogel systems [15] and antioxidant-fortified oleogels incorporating hydrophilic polyphenols [16], the application of a citrus pectin–lecithin emulsion template for constructing camellia oil oleogels loaded with litchi seed polyphenols has not been systematically investigated. Based on this knowledge gap, we hypothesized that a citrus pectin–lecithin emulsion template could provide an effective strategy for constructing structurally stable camellia oil oleogels while improving the stability of incorporated litchi seed polyphenols.

In this study, we developed a novel LSP-loaded camellia oil oleogel system using a citrus pectin–lecithin stabilized emulsion template. We systematically investigated the effect of pectin concentration on the microstructure, rheological behavior, and oxidative stability of the resulting oleogels. Our findings demonstrate that an optimized 3% CP concentration constructs a robust structural framework, providing a sustainable strategy for the high-value utilization of litchi byproducts while offering a healthy lipid alternative for the food and pharmaceutical industries [17].

## 2. Materials and Methods

### 2.1. Raw Materials and Reagents

Litchi seeds were purchased from Linjiejie Food Firm (Genzi Town, Guangzhou, China). Camellia oil was obtained from Zhejiang Jiusheng Oil Tea Technology Co., Ltd. (Jiande, China). Lecithin (food grade, ≥98%) was purchased from Beijing Meirenda Technology Co., Ltd. (Beijing, China). Citrus pectin (degree of esterification, DE = 65%; biotechnology grade) was supplied by Shanghai Yuanye Bio-Technology Co., Ltd. (Shanghai, China). DPPH (AR grade, purity ≥96%) was purchased from Guangzhou Baohui Biotechnology Co., Ltd. (Guangzhou, China). Ethanol (AR grade), methanol (AR grade), and petroleum ether (AR grade) were purchased from Shanghai Macklin Biochemical Co., Ltd. (Shanghai, China). All reagents used were of analytical grade.

### 2.2. Extraction and Purification of LSP

Dried litchi seeds were pulverized and passed through a 40-mesh standard sieve. The extraction and purification of LSP were performed according to previously reported methods with slight modifications [18]. The powder was extracted with 60% (*v*/*v*) ethanol solution at room temperature (25 ± 2 °C) for 48 h. After filtration, the residue was extracted twice more under the same conditions. The filtrates were combined and concentrated using a rotary evaporator (RE-52AA; Shanghai Yarong Biochemical Instrument Factory, Shanghai, China) at 45 °C and −0.08 MPa to obtain crude LSP extract.

The crude LSP extract was loaded onto an AB-8 macroporous adsorption resin (AR grade, Shanghai Macklin Biochemical Co., Ltd., Shanghai, China) at a sample-to-resin ratio of 1:10 (g/g) and a loading flow rate of 1.0 BV/h, and then eluted with a gradient of ultrapure water and 30%, 60%, and 90% ethanol (*v*/*v*) at a flow rate of 2.0 BV/h. The 30% ethanol eluent was collected, concentrated, and freeze-dried to obtain LSP powder. According to the previously reported extraction and purification method [11], the obtained LSP is mainly composed of polyphenolic compounds including procyanidins and catechin derivatives. The total phenolic content of the extract was determined by the Folin–Ciocalteu method and was 338.76 mg gallic acid equivalents (GAE)/g extract. The extracted LSP powder was directly used for subsequent oleogel preparation.

### 2.3. Preparation of LSP-Loaded Oleogels

The oleogels were prepared using an emulsion-templated approach with slight modifications [11]. The oil phase was prepared by dissolving 3.0% (*w*/*w*, based on the oil phase) lecithin in camellia oil, followed by the pre-dispersion of CP in the oil phase under stirring (the concentration was adjusted according to the experimental design). The aqueous phase was 1 mg/mL LSP solution. The two phases were mixed at specific oil-to-water volume ratios and homogenized using a high-shear emulsifier (THF500-18G; Shanghai Tuohe Electromechanical Technology Co., Ltd., Shanghai, China) at 5000 r/min for 2.5 min to obtain the emulsion precursors.

Subsequently, the emulsions were dehydrated in a freeze-dryer (FD-1A-50; Beijing Boyikang Experimental Instrument Co., Ltd., Beijing, China) for 48 h to yield dried gel templates, which were subjected to mechanical shearing at 1200 r/min for 2.5 min to promote structural reorganization into a uniform three-dimensional network. The resulting samples were allowed to equilibrate at room temperature for 24 h to ensure structural stabilization.

To investigate the effect of phase ratios on emulsion performance, nine emulsions containing 3.0% (*w*/*v*) CP were first prepared with different oil-to-water volume ratios (1:9, 2:8, 3:7, 4:6, 5:5, 6:4, 7:3, 8:2, and 9:1, *v*/*v*). After the optimal oil-to-water ratio had been determined, the influence of different CP concentrations (1%, 2%, 3%, and 4%, *w*/*v*) on the characteristics of both the emulsions and the corresponding oleogels was further investigated. The corresponding samples were labeled as CP-1, CP-2, CP-3, and CP-4, respectively, and were utilized for subsequent characterization and analysis. All oleogel samples were independently prepared in triplicate, and each batch was subjected to the subsequent analyses.

### 2.4. Characterization of Emulsion Properties

The types of emulsions (O/W or W/O) prepared at various oil-to-water ratios were identified using the filter paper wetting and cobalt chloride staining method. Briefly, qualitative filter papers (BKMAM; Hunan Bikeman Experimental Equipment Co., Ltd., Changsha, China; 11 cm in diameter) were completely immersed in a 15% (*w*/*v*) cobalt chloride solution (AR grade, Shanghai Macklin Biochemical Co., Ltd., Shanghai, China) for 2 min, then removed and dried using a hot-air blower for 5 min before use. Subsequently, approximately 1.0 mL of freshly prepared emulsion from each group was dropped onto the pretreated filter paper. The emulsion type was determined according to the spreading behavior and color change in the droplets. If the droplets spread rapidly on the filter paper accompanied by a fading of the blue color, the sample was classified as an oil-in-water (O/W) emulsion. Conversely, if the droplets remained localized and the color of the filter paper remained unchanged, the sample was identified as a water-in-oil (W/O) emulsion. All experiments were performed at room temperature (25 ± 1 °C) and independently repeated three times. Additionally, the macroscopic morphology of the emulsions was photographed using the built-in rear camera of a vi-vo S17 smartphone (vivo Mobile Communication Co., Ltd., Dongguan, Guangdong, China) under identical indoor lighting conditions and at a fixed shooting distance of 20 cm to visually present the effects of different oil-to-water ratios and CP addition levels on the emulsion types and appearance characteristics.

### 2.5. Microstructural Characterization of Emulsion Precursors

The micromorphology of the emulsion precursors was observed using both optical microscopy (XSP-63; Ningbo Sunny Instruments Co., Ltd., Ningbo, China) and fluorescence microscopy (DM6000B; Ningbo Sunny Instruments Co., Ltd., Ningbo, China). A thin layer of the emulsion was spread onto a glass slide. The oil phase of the emulsion was specifically stained with a 0.001 g/mL Nile Red solution. At least three representative fields of view were randomly selected for each sample to capture representative images.

Droplet size distribution was determined using a laser diffraction particle size analyzer (Zetasizer Nano ZS; Malvern Panalytical, Malvern, UK). The refractive index and absorption coefficient of the particles were set to 1.520 and 0.1, respectively, while petroleum ether was employed as the dispersant with a refractive index of 1.375. The volume-weighted mean diameter, d_(4,3)_, was used to evaluate the homogeneity and average size of the droplets. All measurements were performed in triplicate.

### 2.6. Characterization of Morphology and Intermolecular Interactions

The micromorphology of dried templates was visualized via CLSM (TCS SP8; Ningbo Sunny Instruments Co., Ltd., Ningbo, China). Sections of approximately 8 μm were stained with Nile Red at an excitation wavelength of 561.6 nm, and 3D models were reconstructed to characterize internal pore structures. The 3D network framework of oleogels was observed using Cryo-SEM (SU8010; Beijing Zhongke Keyi Technology Development Co., Ltd., Beijing, China) at −140 °C and 10 kV. The sample was frozen in liquid nitrogen and then sputtered with gold at low temperature to preserve its original microstructure. Intermolecular interactions were analyzed via FT-IR (IRAffinity-1S; Shimadzu Corporation, Kyoto, Japan). Freeze-dried samples were pressed into KBr pellets at a ratio of 1:100 (*w*/*w*) and 32 scans were accumulated over the range of 400–4000 cm^−1^.

### 2.7. Characterization of Dried Templates and Physical Properties of Oleogels

The texture profile analysis (TPA) was performed according to previously reported methods [11] with slight modifications. The texture profile analysis (TPA) of the dried templates (1 cm thick) was conducted using a texture analyzer (TA-XT2i; Shanghai Baosheng Industrial Development Co., Ltd., Shanghai, China) equipped with a P/6 probe. The pre-test, mid-test, and post-test speeds were set at 5.0, 1.0, and 5.0 mm/s, respectively, to determine hardness, springiness, and cohesiveness.

Oil loss (OL) was measured via centrifugation to assess the oil-binding capacity. Briefly, 4 g of the sample was centrifuged using a high-speed centrifuge (TGL-16M; Shanghai Luxiang Instrument Co., Ltd., Shanghai, China) at 12,000 r/min and 4 °C for 20 min. The OL value was calculated based on the mass difference before and after centrifugation using the following equation:

(1) $$OL\;(\%)=\left(\frac{m_{1}-m_{2}}{m_{1}-m}\right)\times 100\%$$

where *m* is the weight of the empty centrifuge tube; *m*_1_ is the total mass of the tube and oleogels before centrifugation; and *m*_2_ is the mass after removing the precipitated oil phase. Thermal stability was evaluated by treating samples in a water bath at 30, 50, 70, and 90 °C for 30 min, followed by OL determination.

### 2.8. Rheological Measurements

The rheological properties of the oleogels were characterized using a rotational rheometer (MCR 302; Anton Paar, Graz, Austria) equipped with a 40 mm parallel plate system (1000 μm gap). Before testing, samples were equilibrated for 24 h to eliminate residual stress, and the plate edges were sealed with paraffin oil to prevent solvent evaporation.

An amplitude sweep (0.01%~100% strain at 1 Hz) was initially performed to determine the linear viscoelastic region (LVR). Subsequently, frequency sweeps (0.1~10 Hz) were conducted within the LVR to monitor changes in moduli. Steady-state flow behavior was assessed at shear rates ranging from 1 to 100 s^−1^, while thixotropic recovery was evaluated using a three-step shear test (0.1~10~0.1 s^−1^). Finally, a temperature sweep was carried out from 5 to 80 °C at a heating rate of 5 °C/min to record the evolution of the storage modulus (G′) and loss modulus (G″). All measurements were performed in triplicate.

### 2.9. Oxidative Stability and Antioxidant Activity

The antioxidant activity and oxidative stability of the oleogels were evaluated according to previously reported methods with slight modifications [10]. Briefly, 2 mL of the oleogels was mixed with 4 mL of a 1 mmol/L DPPH–methanol solution and incubated in the dark for 40 min. The absorbance was measured at 517 nm using a UV–vis spectrophotometer (752 N; Shanghai INESA Analytical Instrument Co., Ltd., Shanghai, China) to calculate the DPPH radical scavenging rate.

The oil was recovered from the samples through dissolution in petroleum ether, filtration, and subsequent vacuum distillation. The acid value (AV) and peroxide value (POV) were determined according to the Chinese National Standards GB 5009.229-2025 [19] and GB 5009.227-2023 [20], respectively, to evaluate lipid hydrolysis and primary oxidation in the recovered oil phase of the oleogels. The DPPH radical scavenging activity was calculated using the following equation:

(2) $$DPPH\;scavenging\;activity\left(\%\right)=\left(1-\frac{Ai-Aj}{Ao}\right)\times 100\%$$

where *Ao*, *Ai*, and *Aj* represent the absorbance values of the different reaction systems. Specifically, *Ao* is the absorbance of the control (2 mL methanol + 4 mL DPPH solution); *Ai* is the absorbance of the test sample (2 mL sample + 4 mL DPPH solution); and *Aj* is the absorbance of the sample blank (2 mL sample + 4 mL methanol).

### 2.10. Statistical Analysis

All experiments were performed in triplicate. Data are expressed as mean ± standard deviation (SD). Statistical analyses were performed using SPSS 20.0 software. Differences among treatment groups were evaluated by one-way analysis of variance (ANOVA) followed by Duncan’s multiple range test, and *p* < 0.05 was considered statistically significant. Figures were plotted using Origin 2024.

## 3. Results

### 3.1. Characterization of Emulsions Stabilized by Lecithin-CP Complexes

#### 3.1.1. Effect of Oil-to-Water Volume Ratio on the Appearance and Stability of Emulsion

**Figure 1:**
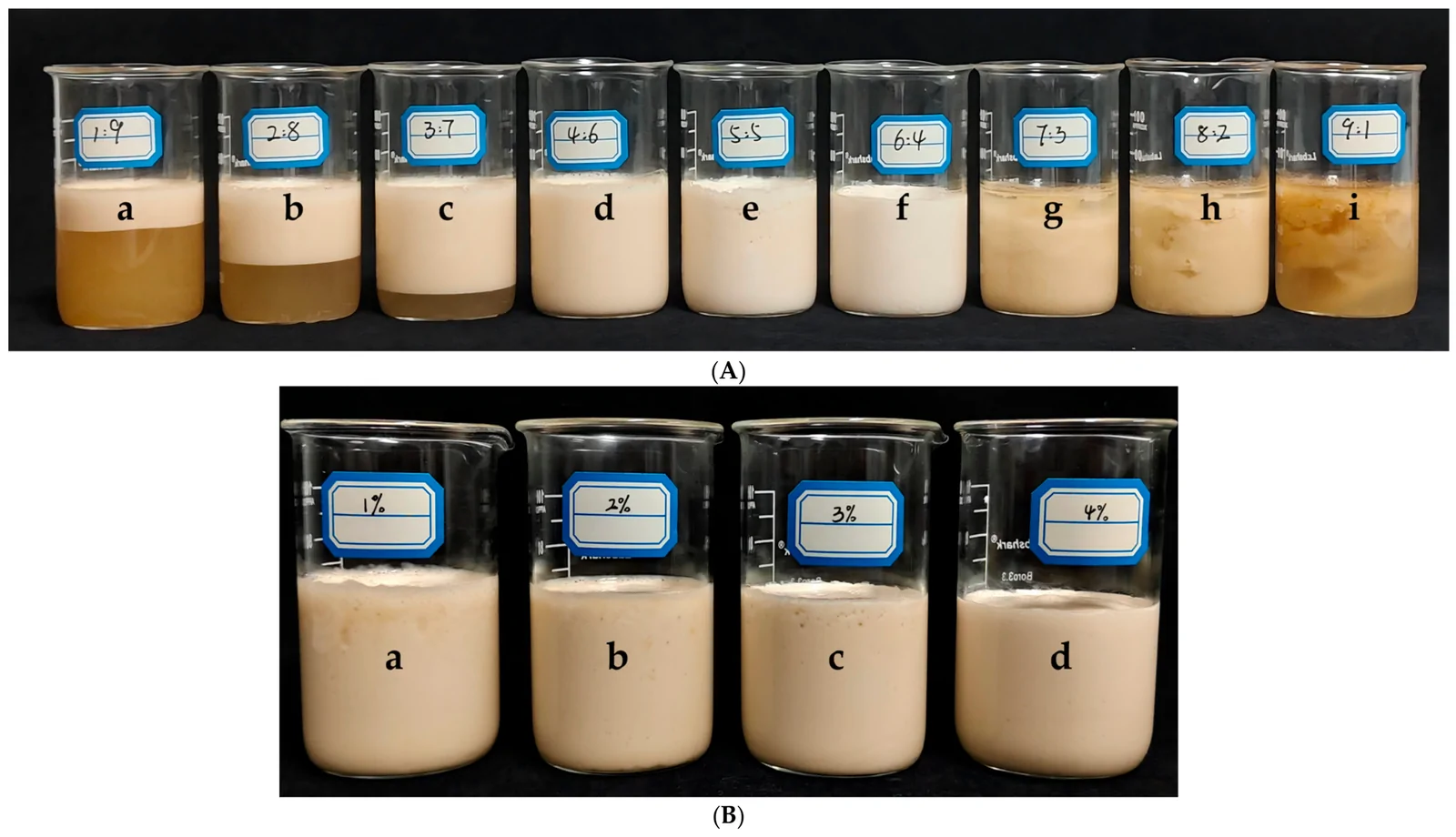
Effects of different preparation conditions on emulsion characteristics: (**A**) Macroscopic appearance of emulsions at various oil-to-water volume ratios; (**B**) Sensory characteristics of emulsions as a function of CP concentration. In panels (**A**), the letters a–i correspond to oil-to-water volume ratios of 1:9, 2:8, 3:7, 4:6, 5:5, 6:4, 7:3, 8:2, and 9:1 (*v*/*v*), respectively. In panel (**B**), at a fixed oil-to-water ratio of 5:5, the letters a–d represent CP concentrations of 1.0%, 2.0%, 3.0%, and 4.0% (*w*/*v*), respectively.

The influence of varying oil-to-water volume ratios on the macroscopic appearance of the emulsions is illustrated in Figure 1A. As the oil phase proportion increased, the stability and phase behavior of the systems exhibited distinct gradient variations.

The emulsions with intermediate oil ratios (5:5 and 6:4) appeared uniform, smooth, and milky white, achieving the maximum emulsion layer volume without detectable phase separation. This superior stability is primarily attributed to the optimized balance between the oil interfacial area and the adsorption capacity of the composite stabilizers. Based on the obtained results and previous reports [11], it is proposed that lecithin and CP may synergistically assemble at the oil–water interface to form a relatively continuous and dense composite interfacial layer, thereby enhancing the steric stabilization between droplets and reducing droplet coalescence.

#### 3.1.2. Effect of CP Concentration on Emulsion Appearance

As shown in Figure 1B, emulsions prepared at low CP concentrations (1.0%~2.0%, *w*/*v*) exhibited poor macroscopic stability and a coarse texture. Increasing the CP concentration to 3.0%~4.0% (*w*/*v*) significantly improved the appearance, yielding milky-white samples with excellent macroscopic homogeneity and no visible surface bubbles. This enhancement is primarily attributed to two factors: (1) the increased viscosity of the continuous phase, which retarded Brownian motion and droplet aggregation and (2) the formation of a robust viscoelastic mechanical barrier at the interface, providing physical obstruction against coalescence [21]. The results indicate that a CP concentration of ≥3.0% is essential for forming uniform emulsions with superior sensory quality.

#### 3.1.3. Identification of Emulsion Types and Phase Inversion Behavior

**Figure 2:**
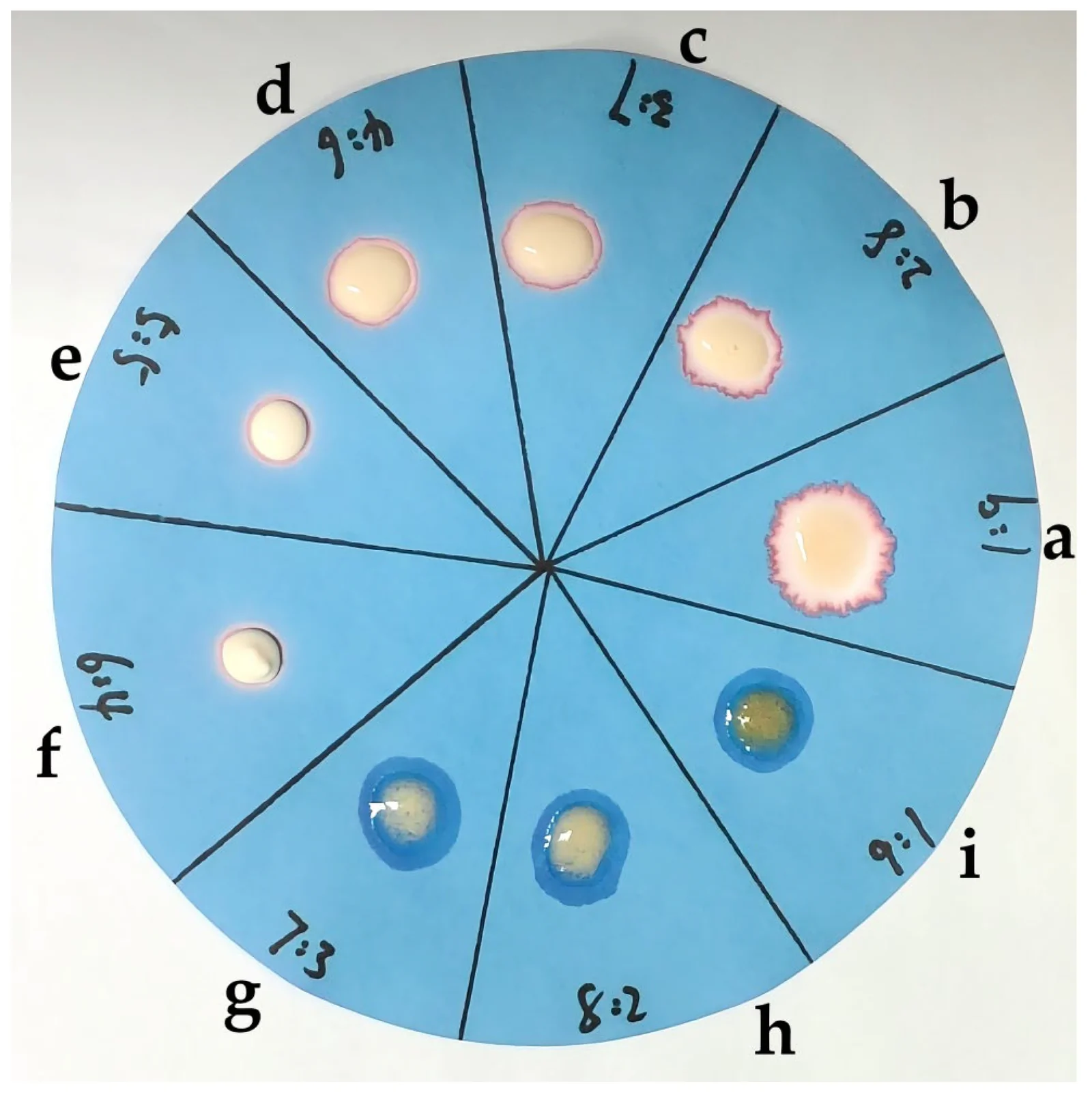
Identification of emulsion types prepared with different oil-to-water volume ratios using the cobalt chloride filter paper wetting-staining method. The oil-to-water volume ratios were (**a**) 1:9, (**b**) 2:8, (**c**) 3:7, (**d**) 4:6, (**e**) 5:5, (**f**) 6:4, (**g**) 7:3, (**h**) 8:2, and (**i**) 9:1 (*v*/*v*). Emulsion type was determined using cobalt chloride-stained filter paper. Rapid spreading of the droplet accompanied by fading of the blue color indicates an oil-in-water (O/W) emulsion, whereas localized droplets with no obvious color change indicate a water-in-oil (W/O) emulsion.

The continuous phase of the emulsions was determined via filter paper wetting-staining assay (Figure 2). Ratios of 1:9~4:6 (a–d) generated O/W emulsions; the 4:6 group displayed slowed droplet diffusion, close to phase inversion boundary.

When oil ratios rose to 5:5~9:1 (e–i), emulsions fully shifted into W/O type. Such phase inversion offers essential formulation guidance for preparing LSP-incorporated oleogels by adjusting oil–water proportions [22].

### 3.2. Microstructure and Droplet Size of Emulsion Precursors

#### 3.2.1. Micromorphology Observation

**Figure 3:**
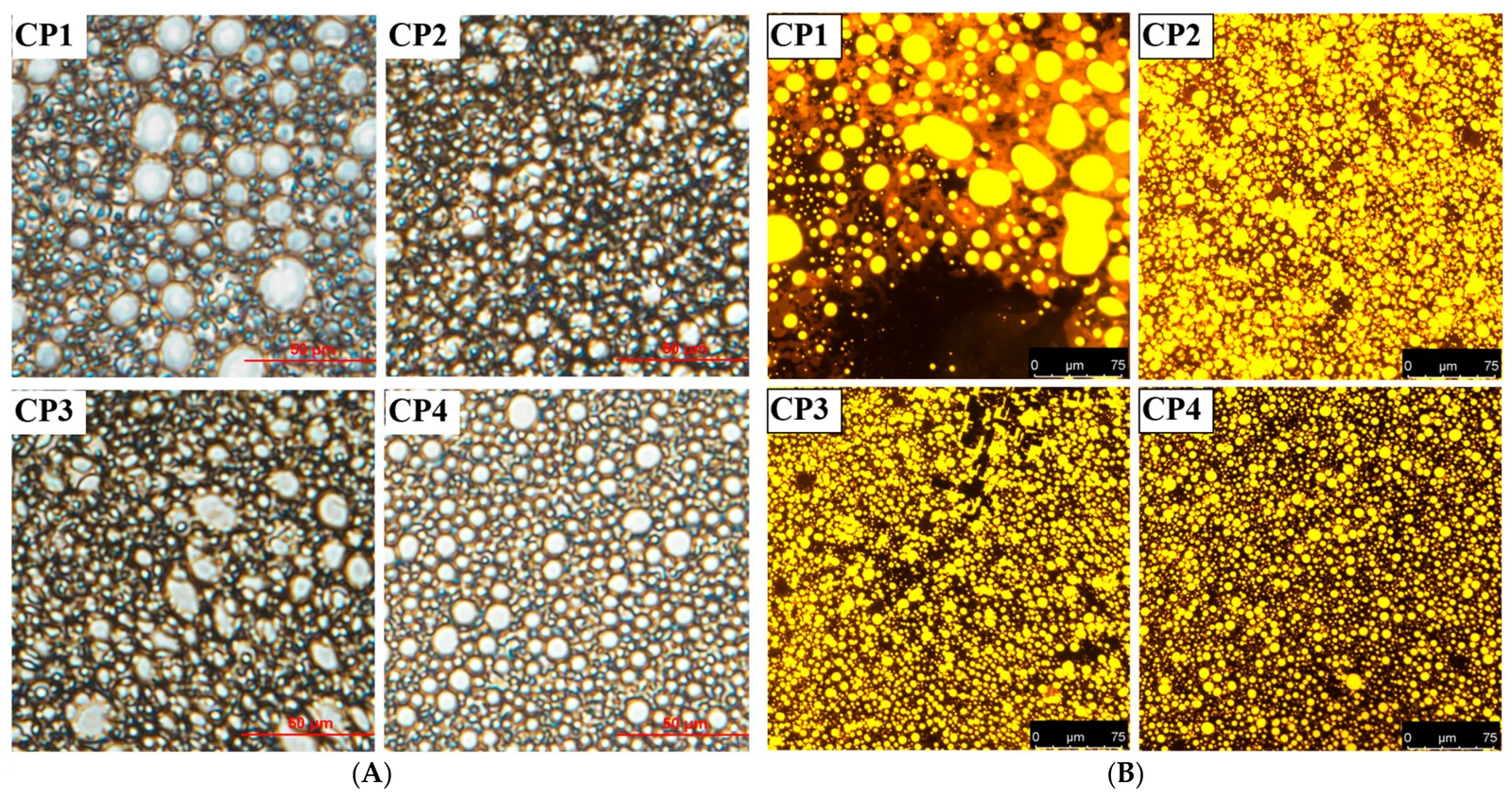
Influence of CP concentration on the microscopic characteristics of emulsions: (**A**) Optical microscopy images and (**B**) fluorescence microscopy images of emulsions at varying CP concentrations. In panels (**A**) and (**B**), CP1–CP4 correspond to CP concentrations of 1.0%, 2.0%, 3.0%, and 4.0% (*w*/*v*), respectively, at a fixed oil-to-water ratio of 5:5. The scale bars for panels (**A**) and (**B**) represent 50 μm and 75 μm, respectively.

Optical microscopy (Figure 3A) revealed the regulatory effect of CP concentration on the emulsion microstructure. At low CP concentration (1.0%, *w*/*v*), the system exhibited high polydispersity with numerous irregular large droplets and aggregates. This indicates that insufficient interfacial coverage at low CP levels resulted in a weak lecithin-based film, which failed to resist droplet coalescence.

As the CP concentration increased to 2.0% (*w*/*v*), the droplet size was significantly refined with reduced aggregation. At higher concentrations (3.0%~4.0%, *w*/*v*), the emulsions reached high uniformity, characterized by densely packed, regular spherical droplets. This improvement is attributed to the synergistic assembly of high-concentration CP and lecithin, forming a dense composite interfacial layer that provides strong steric repulsion and reduced droplet coalescence [23]. Furthermore, fluorescence images (Figure 3B) showed Nile Red-stained oil phase encapsulated within the spherical droplets, consistent with the bright-field observations and confirming the structural stabilization provided by CP.

#### 3.2.2. Mean Droplet Size Analysis

The volume-weighted mean diameter, d_(4,3)_, of the emulsions at various CP concentrations is summarized in Table 1. The data reveal a significant negative correlation between CP concentration and droplet size: as the concentration increased from 1.0% to 4.0%, the d_(4,3)_ value decreased markedly from 8.36 ± 0.59 μm to 3.90 ± 0.24 μm. This grain-refining effect is attributed to the enhanced mechanical barrier at the interface and the increased viscosity of the continuous phase at higher CP levels, which constructed a stable viscoelastic network that hindered Brownian motion and droplet collision frequency [24].

### 3.3. Microstructure and Intermolecular Interactions of the Oleogel System

#### 3.3.1. Micromorphology of Dried Templates

**Figure 4:**
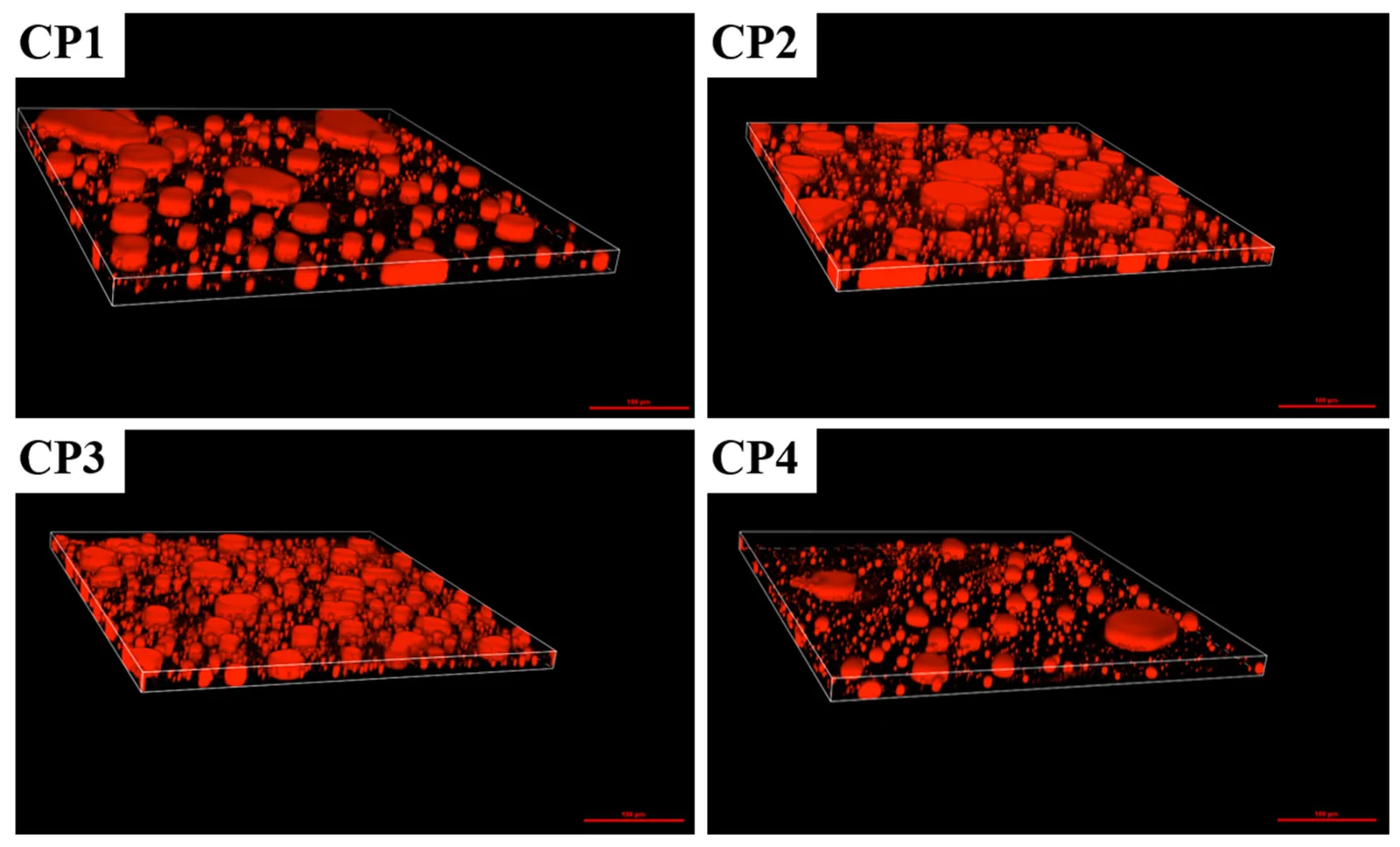
Influence of CP concentration on the microstructures of dried templates. 3D reconstruction images of dried templates at various CP concentrations: (CP1) 1.0%, (CP2) 2.0%, (CP3) 3.0%, and (CP4) 4.0% (*w*/*v*), visualized by confocal laser scanning microscopy (CLSM). The red areas represent the distribution of the oil phase stained with Nile Red. Scale bars are 100 μm.

Confocal laser scanning microscopy (CLSM) was employed to visualize the oil phase distribution within the dried templates (Figure 4). The Nile Red-stained oil phase appeared as red spherical droplets dispersed throughout the matrix, with their size and distribution significantly regulated by the CP concentration. At a low CP concentration (1.0%, *w*/*v*), insufficient interfacial coating failed to restrain droplet merging, resulting in sparse, oversized oil droplets across the matrix.

As the CP concentration increased to 2.0%~3.0%, the droplet size was markedly refined and became more uniform. At a concentration of 4.0%, most oil droplets remained small and well-dispersed, while local droplet aggregation and slight oil leakage were observed. Excess CP triggers incomplete interfacial coverage instead of forming intact protective films. These micromorphological findings correlate with the previously observed enhancement in the macroscopic rigidity of the dried templates, suggesting that efficient oil droplet dispersion may be one of the factors contributing to the observed macroscopic textural properties [25].

#### 3.3.2. Cryo-SEM Analysis of Oleogels

**Figure 5:**
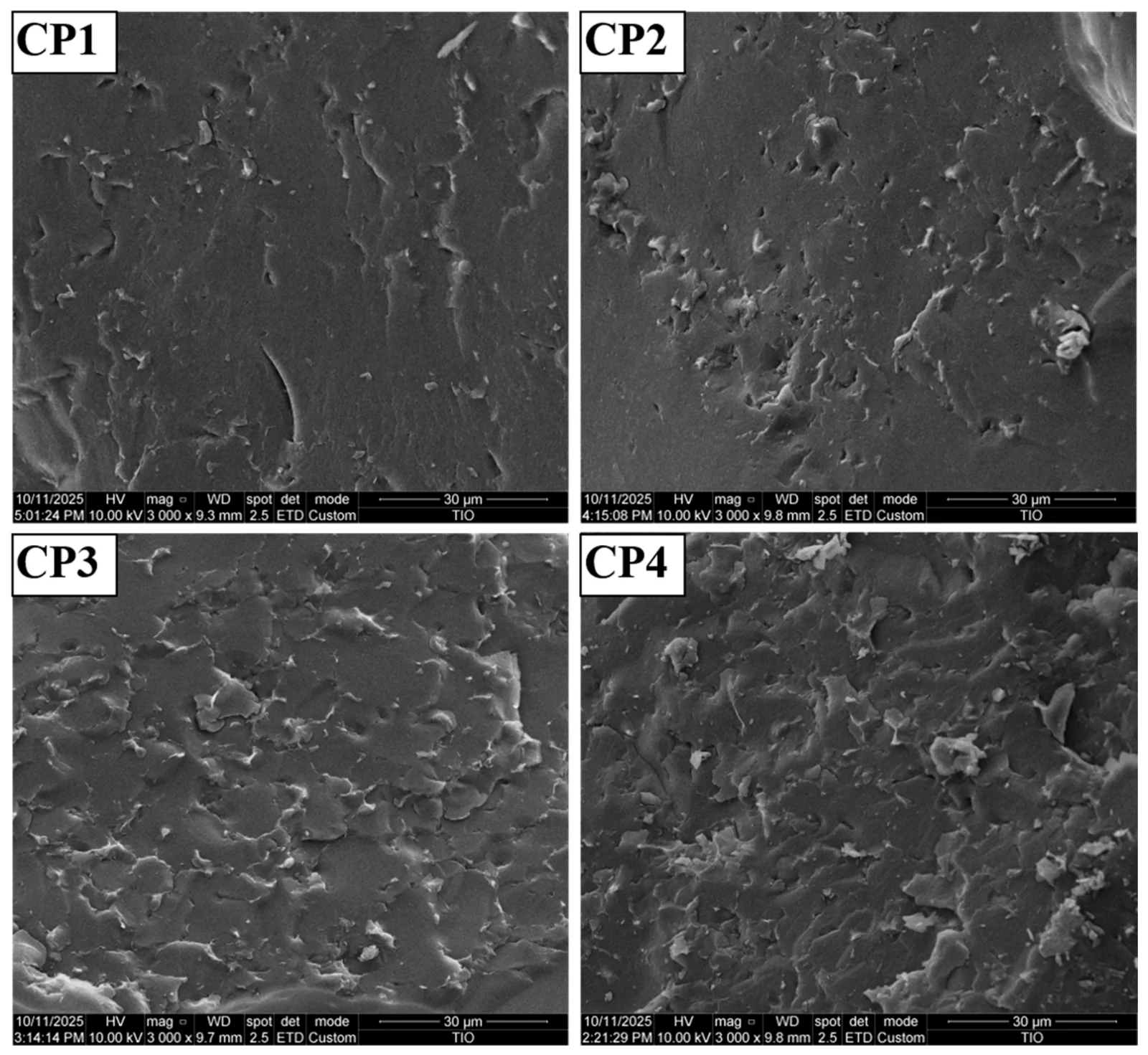
Cryo-SEM images of oleogels at various CP concentrations. Note: CP1–CP4 represent CP concentrations of 1.0%, 2.0%, 3.0%, and 4.0% (*w*/*v*), respectively. The images illustrate the structural evolution of the CP-mediated interfacial network from a smooth state to a highly dense aggregate morphology. Scale bars are 30 μm.

Cryo-SEM revealed the internal three-dimensional network skeleton of the oleogels (Figure 5). At 1.0% CP, the sample contained large aggregated oil lumps and sparse oil droplets alongside smooth matrix regions due to inadequate interfacial strength. Increasing the CP concentration to 2.0%~3.0% yielded denser and more homogeneous networks. Based on the Cryo-SEM observations, pectin and lecithin appeared to form a more continuous supporting structure, which may have contributed to a more uniform distribution of the oil phase.

At 4.0% CP, the microstructure changed into coarse granular aggregates, which may be attributed to excessive entanglement of pectin chains [26]. The resulting compact network structure may have contributed to the improved thermal stability, which is consistent with the macroscopic observations of the oleogels.

#### 3.3.3. FTIR Analysis of Intermolecular Interactions

**Figure 6:**
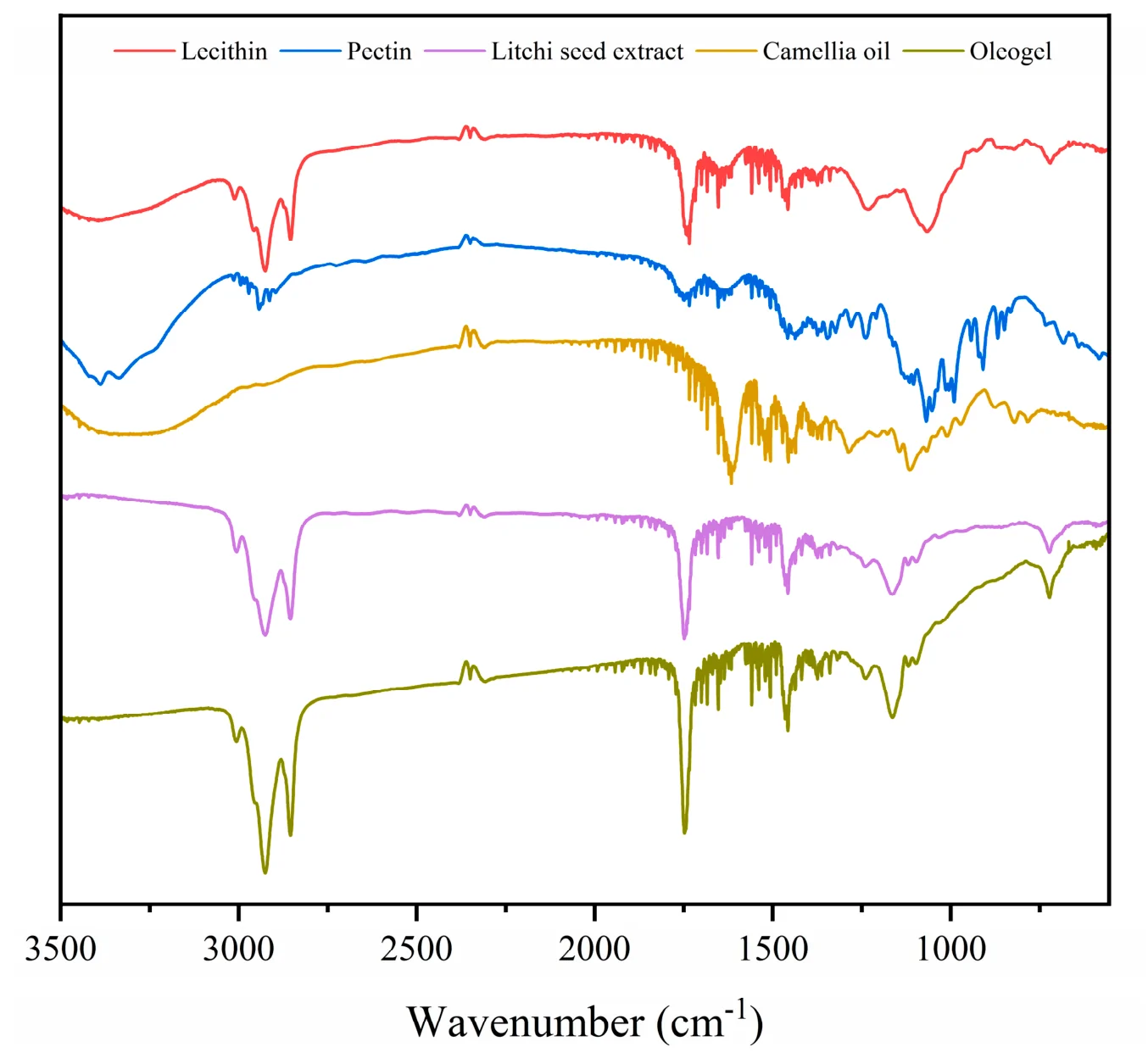
Fourier transform infrared (FTIR) spectra of raw materials and the prepared oleogels. The figure displays the infrared characteristics of lecithin, CP, LSP, camellia oil, and the final oleogels.

Fourier transform infrared (FTIR) spectroscopy was employed to elucidate the molecular driving forces behind oleogel formation (Figure 6). Both camellia oil and the oleogel samples exhibited strong symmetric and asymmetric CH_2_ stretching vibration peaks near 2850 cm^−1^ and 2920 cm^−1^. The consistency in peak shape and position before and after gelation suggests that camellia oil remained physically entrapped within the gel network with-out obvious chemical alteration to its fatty acid chains.

A broad peak corresponding to -OH stretching vibrations appeared in the range of 3200~3500 cm^−1^ for both CP and oleogels, suggesting that hydrogen bonding is the core driving force for constructing the three-dimensional network. Additionally, characteristic peaks near 1740 cm^−1^, 1240 cm^−1^, and 1600 cm^−1^ (aromatic ring vibrations from polyphenols) originated from the individual raw components without significant shifts, indicating that no new chemical bonds were formed during oleogel preparation. Combined with previous reports [27], these FTIR results suggest that the oleogel net-work is primarily stabilized by non-covalent interactions, including hydrogen bonding, hydrophobic interactions, and steric effects. These intermolecular interactions may contribute to the formation of a physically cross-linked network capable of immobilizing camellia oil and facilitating the incorporation of LSP.

### 3.4. Texture and Oil Loss Rate

#### 3.4.1. Textural Properties of Dried Templates

The freeze-dried samples exhibited milky-white, soft-solid characteristics with an internal porous grid structure derived from the emulsion templates, serving as key intermediates for high-performance oleogel fabrication. As shown in Table 2, CP concentration significantly regulated the Texture Profile Analysis (TPA) parameters. As the CP concentration increased from 1.0% to 4.0% (*w*/*v*), the hardness, gumminess, and chewiness showed a significant linear upward trend (*p* < 0.05), with hardness increasing sharply from 0.22 ± 0.04 N to 1.48 ± 0.17 N.

This rigidification is attributed to the thick physical barrier formed by high-concentration CP at the interface and the denser network support system constructed in the continuous phase, which effectively enhanced the templates’ resistance to compressive stress. In contrast, springiness exhibited a phased saturation in response to concentration, while cohesiveness and adhesiveness were less affected. This suggests that the strength of the rigid skeleton is primarily dictated by polysaccharide concentration, whereas deformation recovery capacity depends more on the fundamental interfacial properties of the emulsion template [28].

#### 3.4.2. Response of Oil Loss (OL) to CP Concentration

**Figure 7:**
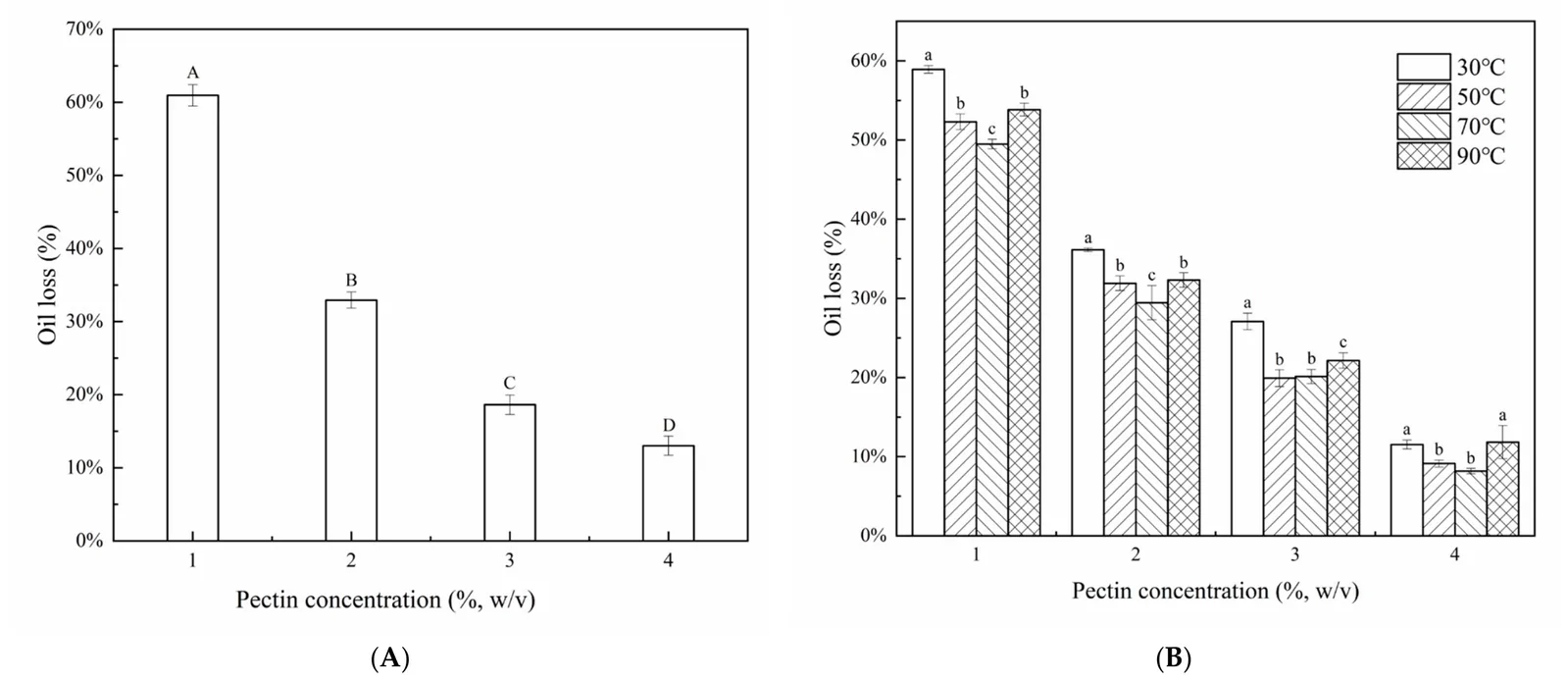
Influence of CP concentration on the physical stability of oleogels. (**A**) Oil retention capacity at various CP concentrations (expressed as centrifugal oil loss); (**B**) Thermal stability of oleogels with different CP concentrations under heat treatments (30 °C~90 °C). Different letters indicate significant differences among different treatments within the same panel (*p* < 0.05).

Oil loss (OL), measured via centrifugation, is a key indicator for evaluating the efficiency of the oleogel microstructure in entrapping the oil phase [29]. As shown in Figure 7A, the OL values exhibited a clear concentration-dependent trend: as the CP concentration increased from 1.0% to 4.0%, the OL value dropped significantly from approximately 60% to 12%, demonstrating exceptional structural stability.

The underlying mechanism is primarily attributed to the synergistic effect of interfacial reinforcement and capillary sequestration. High-concentration CP not only constructed a thick interfacial film to inhibit coalescence during centrifugation but also generated stronger capillary forces through a dense microscopic grid, firmly locking the camellia oil within the gel matrix. These findings are highly correlated with the increasing trend of hardness observed in the textural analysis, collectively confirming that a dense gel skeleton provides the physical foundation for enhancing oil retention capacity.

#### 3.4.3. Thermal Stability and Protection Mechanism

As shown in Figure 7B, the 4.0% CP group exhibited exceptional thermal robustness, with its OL value remaining around 12% even at 90 °C. In contrast, the 1.0% group showed severe oil leakage at high temperatures.

This disparity is primarily due to the robust physical barrier formed by high-concentration CP, which effectively inhibited thermally induced coalescence. Even as oil viscosity decreased with heating, the dense network maintained its structural integrity and physical obstruction. These results confirm that optimizing CP concentration significantly enhances the stability of oleogels in thermal processing environments [30].

### 3.5. Rheological Behavior of Oleogels

#### 3.5.1. LVR and Strain Sensitivity

**Figure 8:**
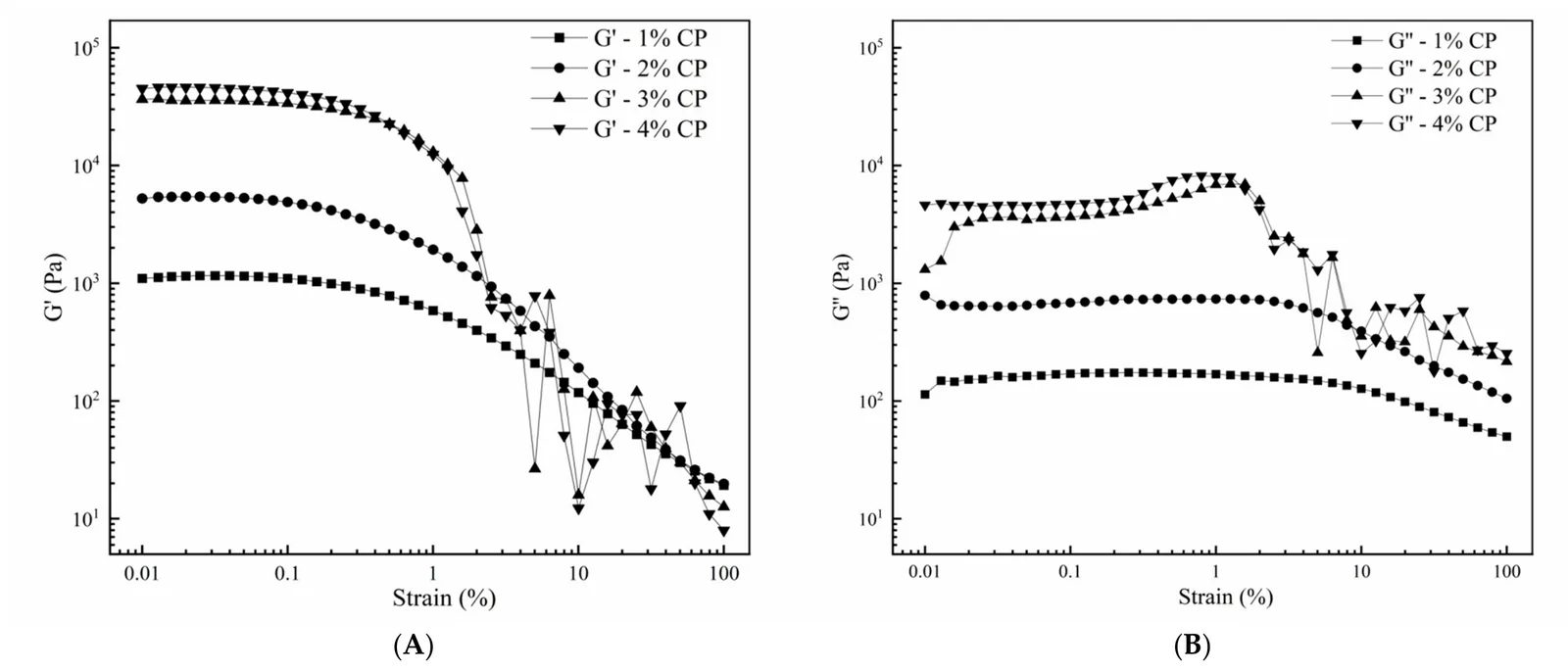
Strain sensitivity and LVR curves of oleogels. Strain sweeps (0.01%~100%) were performed at a fixed frequency of 1 Hz: (**A**) storage modulus (G′) and (**B**) loss modulus (G″). The figure illustrates the regulatory effect of different CP concentrations (1%~4%, *w*/*v*) on the structural stability of the oleogels.

Strain sweeps (Figure 8) were conducted to evaluate the structural stability of the oleogels [31]. In the low-strain region (γ < 1%), both storage modulus (G′) and loss modulus (G″) maintained a constant plateau, indicating that the systems remained within the LVR with intact networks.

As the CP concentration increased from 1% to 4%, the G′ values within the LVR exhibited an order-of-magnitude increase; specifically, the G′ of the 4% CP group was approximately 100-fold higher than that of the 1% group. This confirms that high CP levels significantly enhance mechanical rigidity by constructing a denser three-dimensional network. Beyond the critical strain (γ > 1%), G′ and G″ dropped sharply, signaling structural collapse. Notably, the higher-concentration groups showed a steeper decline in moduli upon yielding, reflecting the more brittle fracture characteristics of the highly rigid networks under strong shear.

#### 3.5.2. Dynamic Frequency Response and Viscoelastic Characteristics

**Figure 9:**
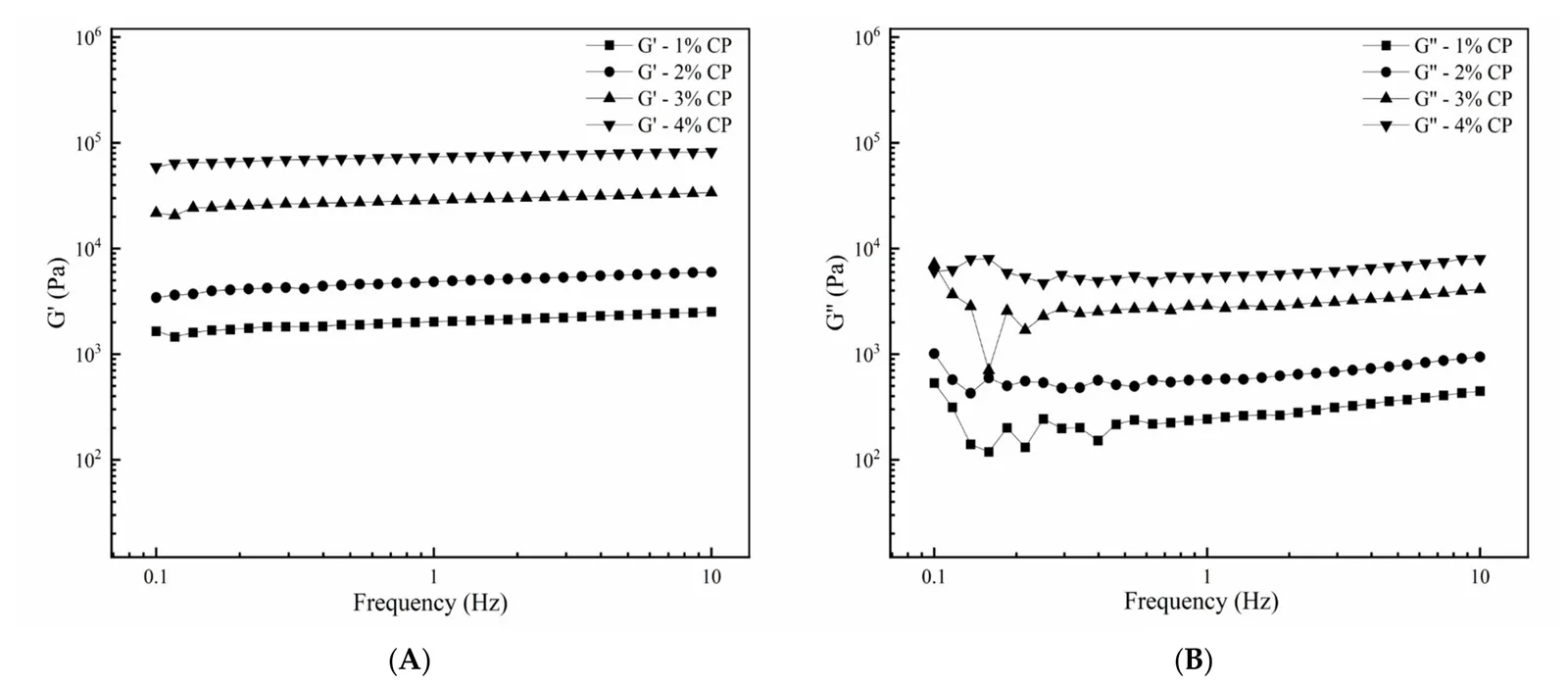
Frequency dependence of the viscoelasticity of oleogels. Frequency sweeps (0.1~10 Hz) were conducted at a fixed strain of 0.01%: (**A**) storage modulus (G′) and (**B**) loss modulus (G″). The figure illustrates the regulatory effect of different CP concentrations (1%~4%, *w*/*v*) on the viscoelastic properties of the oleogels.

Frequency sweeps (Figure 9) were performed to elucidate the microstructural stability and viscoelastic nature of the oleogels. Within the range of 0.1~10 Hz, the G′ of all samples exhibited minimal frequency dependence and remained consistently higher than G″ (G′ > G″), demonstrating typical solid-like behavior and strong gel characteristics [32].

As the CP concentration increased, both G′ and G″ shifted significantly upward, attributed to the increased density of physical cross-linking points between CP molecules and at the oil–water interface. The slight fluctuations observed in the 1% CP group at low frequencies (0.1~0.5 Hz) reflect network relaxation behavior at low concentrations. In contrast, the stable curves of the high-concentration groups (3%~4% CP) confirm that the dense pectin network provides superior resistance to dynamic shear.

#### 3.5.3. Steady-State Flow Behavior and Shear-Thinning Characteristics

**Figure 10:**
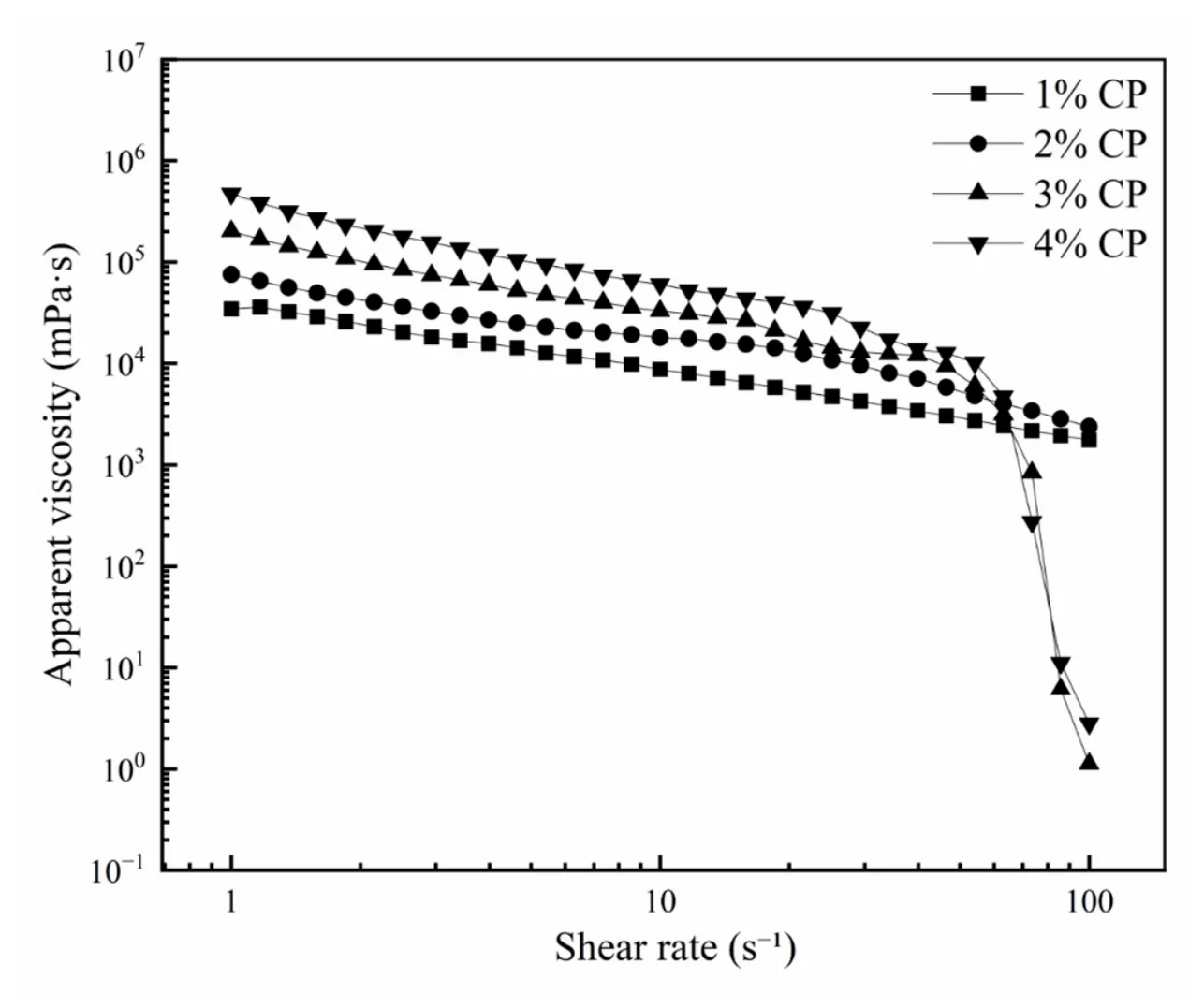
Apparent viscosity of oleogels as a function of shear rate. Steady-state shear sweeps (1~100 s^−1^) were performed at 25 °C. The figure illustrates the regulatory effect of different CP concentrations (1%~4%, *w*/*v*) on the flow behavior and apparent viscosity of the oleogels. Data are representative of three independent replicates.

The apparent viscosity of the oleogels as a function of shear rate is shown in Figure 10. All samples exhibited significant shear-thinning behavior, where apparent viscosity decayed exponentially with increasing shear rate (1~100 s^−1^). This non-Newtonian fluid characteristic arises from the external shear force overcoming the attractive forces between oil droplets, leading to the disintegration of flocculated structures and the oriented flow of liquid oil.

At the same shear rate, the high-concentration CP groups displayed higher viscosity, indicating that the robust network structure significantly enhanced the system’s oil-retention capacity. However, the high-concentration groups also showed a more pronounced drop in viscosity under strong shear, further confirming the high sensitivity of their network structures to mechanical shear.

#### 3.5.4. Thixotropy and Structural Recovery Capacity

**Figure 11:**
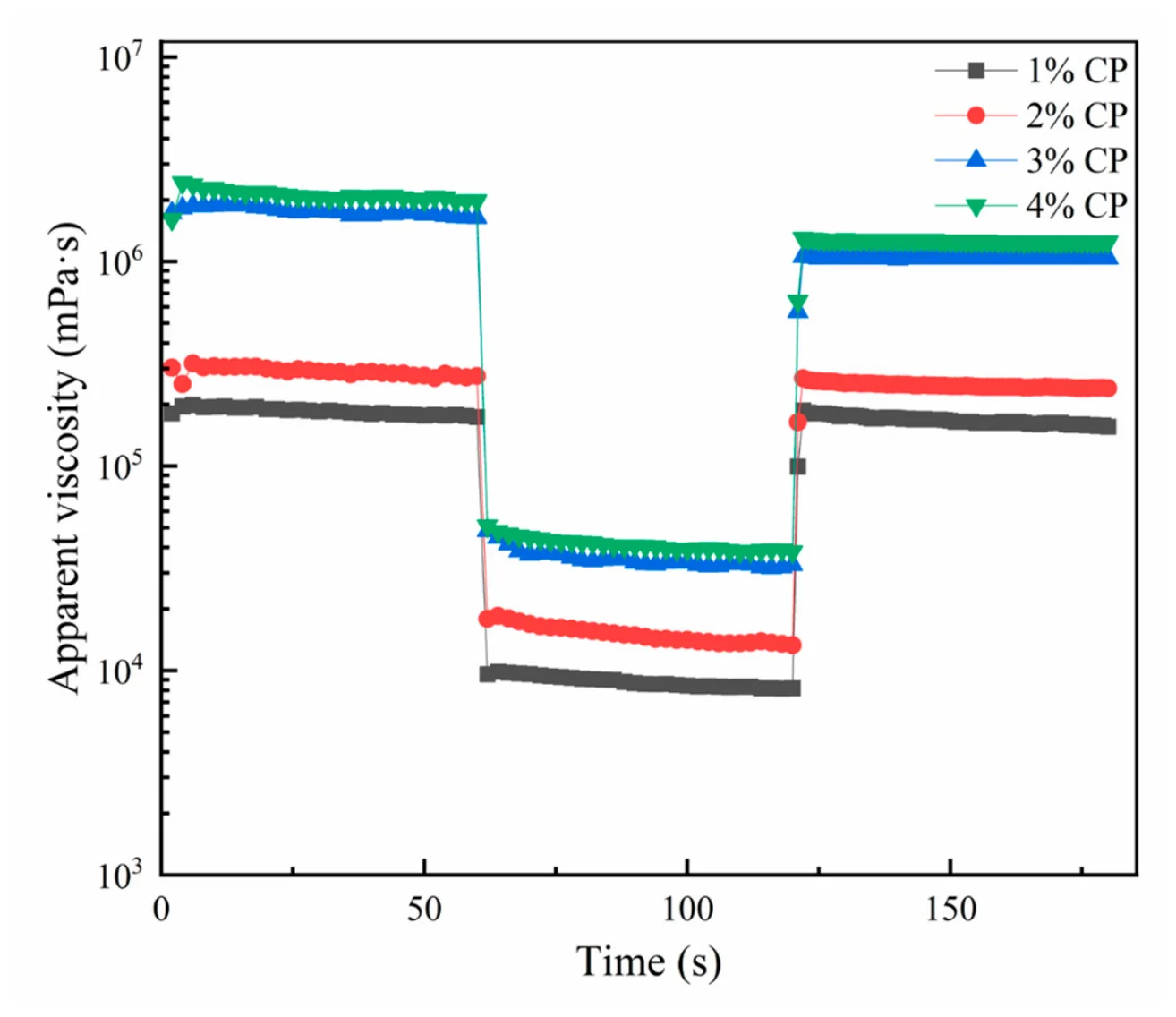
Time-dependent thixotropic recovery curves of oleogels. Three-step shear tests (low/high/low shear rates) were performed at 25 °C. The figure illustrates the regulatory effect of different CP concentrations (1%~4%, *w*/*v*) on the structural recovery and thixotropic properties of the oleogels. Data are representative of three independent replicates.

A three-step shear test was employed to evaluate the self-assembly recovery of the oleogels after extreme shear (Figure 11). All samples exhibited excellent thixotropic recovery: viscosity dropped instantaneously upon a sudden increase in shear rate and recovered rapidly once the external force was removed.

CP concentration played a central role in regulating the recovery efficiency. Both the initial viscosity and the recovered plateau values of the 4% CP group were significantly higher than those of the 1% group. This indicates that the three-dimensional skeleton formed by high-concentration CP not only possesses higher initial strength but also stores more elastic energy. Consequently, it can more effectively drive the physical restoration of the network structure upon shear removal, achieving superior structural retention.

#### 3.5.5. Thermal Sensitivity and Robustness Evaluation

**Figure 12:**
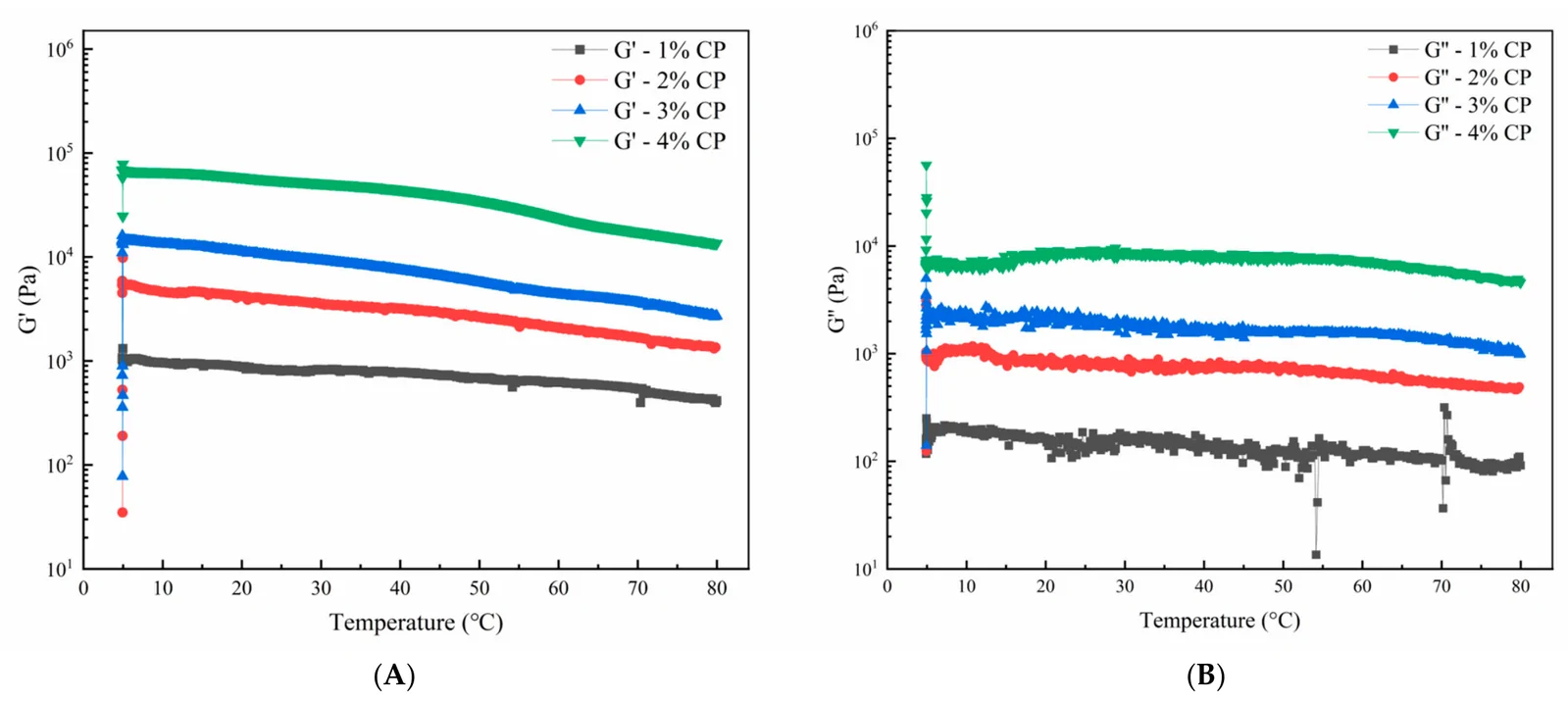
Temperature dependence of the dynamic viscoelastic moduli of oleogels. Temperature sweeps (5~80 °C) were performed at a fixed frequency of 1 Hz and a strain of 0.01%: (**A**) storage modulus (G′) and (**B**) loss modulus (G″). The figure illustrates the regulatory effect of different CP concentrations (1%~4%, *w*/*v*) on the thermal robustness and structural stability of the oleogels.

Temperature sweeps (Figure 12) were conducted to evaluate the physical integrity of the oleogels during heating [33]. Throughout the 5~80 °C heating cycle, both G′ and G″ of all samples remained highly stable (G′ > G″, 102~105 Pa) without detectable melting peaks or phase transition points. This indicates exceptional thermal robustness, where the system maintains its gel state without undergoing sol–gel transition at elevated temperatures.

This non-thermal-sensitive characteristic is attributed to the macromolecular physical network of CP, which relies on non-covalent interactions (e.g., hydrogen bonding and hydrophobic interactions), unlike traditional small-molecule wax-based oleogels. This robust framework provides theoretical support for the application of this system in thermal processing environments.

### 3.6. Quality Parameters and Oxidative Stability

Quality parameters of camellia oil and its oleogels exhibited significant differences (*p* < 0.05, Table 3). The DPPH radical scavenging activity of the oleogels (83.07% ± 0.06%) was significantly higher than that of pure oil (73.89% ± 0.04%). These results suggest that the incorporation of LSP enhanced the DPPH radical scavenging capacity of the oleogels.

Consistently, the peroxide value (POV) of the oleogels (0.92 ± 0.01 mmol/kg) was significantly lower than that of the oil (1.27 ± 0.01 mmol/kg). The lower peroxide value suggests improved resistance to primary lipid oxidation was attributed to the dense three-dimensional network, which acts as a physical barrier limiting the diffusion of pro-oxidants (e.g., oxygen) into the oil matrix, thereby inhibiting hydroperoxide formation [16]. Notably, the POV was far below the limit (19.7 mmol/kg) specified in the National Food Safety Standard (GB 2716-2018) [34], confirming its superior quality and safety.

However, the acid value (AV) of the oleogels (1.85 ± 0.03 mg KOH/g) was higher than that of the pure oil (0.13 ± 0.01 mg KOH/g). This phenomenon was not caused by lipid hydrolysis but rather by the “titratable acidity” of the loaded polyphenols (e.g., syringic acid). These compounds contain phenolic hydroxyl or carboxyl groups that react with the alkali titrant during testing, leading to elevated AV readings.

Overall, the improved DPPH radical scavenging activity and the reduced peroxide value suggest that the oleogel system exhibited enhanced oxidative stability. This improvement may be attributed to the radical scavenging ability of LSP together with the physical protection provided by the gel network.

## 4. Discussion

Based on the above results, the formation of LSP-loaded camellia oil oleogels depends on the synergistic effect among CP, lecithin, camellia oil and LSP. This system first forms the initial structure through an emulsion template, and then completes oil phase immobilization and network reconstruction through freeze-drying and shearing. The transition from O/W to W/O emulsion with increasing oil fraction suggests that the balance between dispersed phase volume and interfacial stabilization capacity is critical. At lower oil ratios, insufficient oil phase may limit the formation of a continuous lipid domain, whereas excessive oil content may exceed the emulsification capacity of lecithin, leading to phase inversion. Therefore, the stability of the emulsion stage has an important influence on the microstructure and macroscopic properties of the final oleogels [6].

CP concentration is a key factor regulating emulsion stability and oleogel structural properties. The 4.0% CP was beneficial for forming emulsion precursors with smaller droplet size and more uniform distribution, while the 3.0% CP oleogels showed better comprehensive performance. This indicates that an appropriate CP concentration can achieve a good balance among emulsion stability, network uniformity and oil phase immobilization. At low CP concentration, insufficient interfacial coverage made oil droplets prone to aggregate, resulting in a loose network structure. As CP concentration increased, pectin chain entanglement, steric hindrance and the CP–lecithin interfacial composite structure were enhanced together, making oil droplets more stably fixed in the gel network, thereby improving oil-holding capacity and thermal stability [35]. However, excessive CP concentration may increase the viscosity of the emulsion system, which could restrict droplet movement during homogenization and influence droplet size distribution. After freeze-drying, excessive polymer content may also result in a more rigid and heterogeneous network, which may explain why the 4.0% CP oleogels did not exhibit the best overall performance.

The microstructure and rheological results further confirmed this structural strengthening effect. With the increase in CP concentration, the storage modulus and apparent viscosity of the oleogels increased, indicating that a stronger elastic network was formed in the system. During frequency and temperature sweeps, G′ was always higher than G″, indicating that the system had typical solid-like gel characteristics and maintained good structural stability during heating. This stability indicates that the oleogel system can maintain oil phase binding ability under certain heat treatment conditions, showing potential application value as a structured lipid system. Similar rheological behavior has also been reported in previous emulsion-templated oleogel systems, where the predominance of G′ over G″ indicated the formation of stable viscoelastic gel networks [11].

FTIR results showed that no new chemical bonds were formed during oleogel formation, and the system was mainly stabilized by non-covalent interactions such as hydrogen bonding, hydrophobic interaction and steric hindrance. During freeze-drying, the removal of water reduced the intermolecular distance among citrus pectin, lecithin, and litchi seed polyphenols, facilitating hydrogen bonding and other non-covalent interactions that promoted the self-assembly of a physically cross-linked three-dimensional network. These results suggest that CP mainly contributed to the structural framework of the network, while lecithin stabilized the oil–water interface. Camellia oil was physically immobilized within the three-dimensional network, and LSP was successfully incorporated into the oleogel without undergoing chemical modification.

The oleogels showed obvious shear-thinning and thixotropic recovery behaviors. Under external force, the gel network could be temporarily destroyed, giving the system a certain fluidity. Once the external force was removed, the viscosity could recover, indicating some self-recovery ability of the network structure. This characteristic is beneficial for its use in spreadable lipid foods, functional oil carriers or local application systems [32].

In terms of oxidative stability, the addition of LSP improved the free radical scavenging ability of the oleogels and reduced the peroxide value. This may be because LSP provides chemical antioxidant activity, while the three-dimensional network constructed by CP–lecithin limits the diffusion of oxygen and pro-oxidant factors into the oil phase. Therefore, the antioxidant stability of this system comes from the synergistic effect of polyphenol antioxidation and physical protection by the gel network [36].

It should be noted that the higher acid value of oleogels than that of pure camellia oil does not necessarily indicate enhanced lipid hydrolysis. The phenolic hydroxyl groups or carboxyl groups in LSP may interfere with the acid value determination, thereby increasing the apparent acid value. Therefore, the oxidative quality of polyphenol-containing oleogels should be comprehensively evaluated by DPPH, POV and active component stability, rather than relying only on a single acid value index.

Overall, this study established the relationship among CP concentration, emulsion template stability, oleogel microstructure and functional properties. CP and lecithin improved the oil-holding capacity, rheological properties and thermal stability of oleogels through interfacial complexation and network construction, while LSP further provided antioxidant function to the system. This system provides a new approach for the structured utilization of camellia oil and the high-value development of litchi seed by-products [37].

Although the developed oleogels exhibited promising physicochemical properties and oxidative stability, several limitations should be acknowledged. First, this study mainly focused on physicochemical characterization under laboratory conditions, while the performance of the developed oleogels in real food systems remains to be evaluated. Second, long-term storage stability and digestion behavior were not investigated. In addition, the molecular interactions among citrus pectin, lecithin, and litchi seed poly-phenols were inferred based on the obtained results and require further verification using advanced analytical techniques. Future studies will address these aspects to further explore the application potential of this oleogel system.

## 5. Conclusions

This study successfully constructed LSP-loaded camellia oil oleogels using a CP–lecithin stabilized emulsion template. The optimized oleogel was mainly composed of approximately 91.9% camellia oil, 5.0% lecithin, 3.0% CP and 0.10% LSP, indicating that this system could achieve effective gelation of a high-oil-content system with low amounts of structuring agents and active components. The results showed that CP concentration was a key factor in regulating emulsion stability and the physical properties of oleogels. Specifically, 4.0% CP was beneficial for forming emulsion precursors with smaller droplet size and more uniform distribution, while 3.0% CP oleogels showed better comprehensive performance, achieving a good balance among network uniformity, oil-holding capacity, thermal stability and antioxidant ability.

FTIR results suggested that the oleogel network was primarily stabilized by non-covalent interactions without the formation of new chemical bonds. Hydrogen bonding, hydrophobic interactions, and steric effects were considered to be possible contributors to network formation based on the FTIR results and the relevant literature. The loading of LSP further enhanced the DPPH radical scavenging activity and oxidative stability of the oleogels, which may be attributed to the free radical scavenging ability of litchi seed polyphenols and the physical protection of the oil phase by the CP–lecithin gel network. In summary, LSP-loaded camellia oil oleogels have high oil content, structural stability and oxidative protection ability, providing a reference for the structured utilization of camellia oil [38] and the high-value utilization of litchi seed by-products [39].

## 6. Patents

A patent application based on this work is currently being prepared for submission.

## Figures and Tables

**Table 1:** Droplet size of emulsions at different pectin concentrations.

Sample	Pectin Concentration (%, *w*/*v*)	d_(4,3)_ μm
CP1	1%	8.36 ± 0.59 ^a^
CP2	2%	6.85 ± 0.15 ^b^
CP3	3%	4.56 ± 0.11 ^c^
CP4	4%	3.90 ± 0.24 ^d^

D_(4,3)_ represents the volume-weighted mean diameter. Data are presented as mean ± standard deviation (n = 3). Values within the same column followed by different superscript letters (a–d) are significantly different (*p* < 0.05).

**Table 2:** Texture profile analysis (TPA) parameters of dried emulsion templates with varying pectin concentrations.

Samples	Hardness (N)	Springiness (N·mm)	Cohesiveness	Adhesiveness (N·mm)	Gumminess (N)	Chewiness (mJ)	Resilience
CP1	0.22 ± 0.04 ^a^	0.91 ± 0.18 ^a^	0.39 ± 0.07 ^a^	0.04 ± 0.01 ^a^	0.08 ± 0.00 ^a^	0.08 ± 0.02 ^a^	0.01 ± 0.01 ^a^
CP2	0.57 ± 0.09 ^b^	1.51 ± 0.03 ^b^	0.26 ± 0.06 ^b^	0.06 ± 0.01 ^b^	0.15 ± 0.03 ^b^	0.22 ± 0.05 ^b^	0.05 ± 0.01 ^b^
CP3	0.97 ± 0.19 ^c^	1.47 ± 0.64 ^b^	0.26 ± 0.04 ^b^	0.06 ± 0.01 ^b^	0.24 ± 0.02 ^c^	0.37 ± 0.18 ^c^	0.07 ± 0.02 ^c^
CP4	1.48 ± 0.17 ^d^	1.84 ± 0.60 ^c^	0.21 ± 0.02 ^c^	0.04 ± 0.00 ^a^	0.31 ± 0.03 ^d^	0.56 ± 0.16 ^d^	0.05 ± 0.01 ^d^

Values are expressed as mean ± standard deviation (n = 3). Different lowercase letters in the same column indicate significant differences (*p* < 0.05).

**Table 3:** Quality parameters of camellia oil and camellia oil-based oleogels.

Samples	DPPH Scavenging Activity (%)	Acid Value (mg KOH/g Oil)	Peroxide Value (mmol/kg)
Camellia oil	73.89 ± 0.04 ^b^	0.13 ± 0.01 ^b^	1.27 ± 0.01 ^b^
Oleogels	83.07 ± 0.06 ^a^	1.85 ± 0.03 ^a^	0.92 ± 0.01 ^a^

Values are expressed as mean ± SD (n = 3). Different lowercase superscripts in the same column indicate significant differences (*p* < 0.05).

## Data Availability

The original contributions presented in this study are included in the article. Further inquiries can be directed to the corresponding authors.

## Abbreviations

The following abbreviations are used in this manuscript:

AV	Acid Value
CLSM	Confocal Laser Scanning Microscopy
CP	Citrus Pectin
Cryo-SEM	Cryogenic Scanning Electron Microscopy
FTIR	Fourier Transform Infrared Spectroscopy
LC-MS	Liquid Chromatography-Tandem Mass Spectrometry
LSP	Litchi Seed Polyphenols
LVR	Linear Viscoelastic Region
OL	Oil Loss
POV	Peroxide Value
TPA	Texture Profile Analysis
